# Comprehensive study of rice YABBY gene family: evolution, expression and interacting proteins analysis

**DOI:** 10.7717/peerj.14783

**Published:** 2023-02-24

**Authors:** Ting Zhang, Anqi Wu, Xiaosong Hu, Qiyu Deng, Ziyi Ma, Lina Su

**Affiliations:** 1 College of Bioengineering, Jingchu University of Technology, Jingmen, Hubei, China; 2 Hubei Engineering Research Center for Specialty Flowers Biological Breeding, Jingchu University of Technology, Jingmen, Hubei, China; 3 National Key Laboratory of Crop Genetic Improvement, Huazhong Agricultural University, Wuhan, Hubei, China

**Keywords:** OsYABBYs, Expression profile, Molecular docking simulation, Protein-protein interaction

## Abstract

As plant-specific transcription regulators, YABBYs are involved in plant growth, development and stress responses. However, little information is available about genome-wide screening and identification of OsYABBY-interacting proteins. In this study, phylogenetic relationship, gene structure, protein structure and gene expression profile of eight *OsYABBY*s were carried out, all of which indicated that *OsYABBY*s were involved in different developmental processes and had functional differentiation. More importantly, PPI (protein-protein interaction) analysis and molecular docking simulation predicted that WUSCHEL-related homeobox (WOX) proteins might be interacting proteins of OsYABBYs. Yeast two-hybrid (Y2H) and luciferase complementation imaging assays (LCI) further confirmed that OsYABBYs (except for OsYABBY7) could interact with OsWOX3A *in vitro* and *in vivo*. In addition, OsYABBY3 and OsYABBY5 also could interact with OsWUS. Taken together, our results provided valuable information for further elucidating OsYABBYs regulation mechanism in improving rice performance.

## Introduction

Plants, even more than animals, must respond to adverse environmental conditions, and make appropriate adjustments in their growth and development in order to survive and reproduce. During their life cycle, plants have evolved various mechanisms to perceive challenges. Among these, transcriptional regulators (TRs) play vital roles in activating or repressing key gene expression (Riechmann et al., 2000; Johnson et al., 2007). *YABBY*s, as plant-specific gene family, encode a family of TRs containing two conserved domains: a C2C2-type zinc finger domain in the N-terminal region, and a helix-loop-helix YABBY domain (helix-loop-helix motif) in the C-terminal region (Bowman, 2000; Jang et al., 2004). Most members of *YABBY* family are expressed in a polar manner in lateral organs and involved in developmental processes such as leaf growth, floral organ development, leaf margin establishment and polarity maintenance (Hou, Lin & Hou, 2020; Kang et al., 2022; She et al., 2022; Yang et al., 2022).

In *Arabidopsis thaliana*, six *YABBY* genes have been identified: *FILAMENTOUS FLOWER* (*FIL*), *CRABS CLAW* (*CRC*), *INNER NO OUTER* (*INO*), *YABBY2* (*YAB2*), *YABBY3* (*YAB3*), and *YABBY5* (*YAB5*) (Sieber et al., 2004; Lee et al., 2005a). *FIL*, *YAB2*, *YAB3*, and *YAB5* are considered to be involved in vegetative organ development (Soundararajan et al., 2019). In establishment of adaxial-abaxial pattern of leaf early development, FIL and YAB3 directly regulate *KAN1* (*KANADI1*) and *ARF4* (*AUXIN RESPONSE FACTOR 4*) expression, which in turn set up a positive feedback loop. In addition, FIL and YAB3 interact with transcriptional co-repressors such as LEUNIG (LUG) and the closely related LEUNIG_HOMOLOG (LUH) to form a repressive complex, which negatively regulates potential adaxial-promoting factors (Stahle et al., 2009; Bonaccorso et al., 2012). By contrast, *CRC* and *INO* expression are restricted to reproductive organs (Eckardt, 2010). *CRC* is activated by some MADS proteins such as APETALA3, PISTILLATA, AGAMOUS, and SEPALLATA to regulate nectary and carpel development (Lee et al., 2005a; Gross, Broholm & Becker, 2018). *INO* is specifically expressed in the abaxial region of the ovule primordium, and involved in the formation and asymmetric growth of the outer integument (Gallagher & Gasser, 2008). Recent studies suggest that *INO* reduces the early iron storage in seeds by repressing the expression of *NATURAL RESISTANCE-ASSOCIATED MACROPHAGE PROTEIN 1* (*NRAMP1*) (Sun et al., 2021). In cucurbits, wintersweet and petunia, *CRC* regulates carpel and nectary development, and displays similar expression patterns to *AtCRC* (Lee et al., 2005b; Li et al., 2018; Morel et al., 2018; Zhang et al., 2022). So far, most members of *YABBY* gene families in dicotyledonous plants are reported to participate in the determination of abaxial cell fates and promote leaf abaxial development, while maize *CRC-*like genes *drooping leaf1* (*drl1*) and *drooping leaf2* (*drl2*) do not exhibit polar expression profiles in meristems (Strable et al., 2017; Strable & Vollbrecht, 2019).

Compared with *Arabidopsis*, rice genome comprises eight *OsYABBY* genes (Toriba et al., 2007). Knockout/knockdown or ectopic expressing some *OsYABBY*s do not cause changes in the polarity of the lateral organs, indicating that these *YABBY*s may have different functions between rice and *Arabidopsis* (Jang et al., 2004; Yamaguchi et al., 2004). Rice *DL* (*DROOPING LEAF*), an orthologous gene of *AtCRC*, is required for flower development and leaf vein formation (Ohmori et al., 2011; Sugiyama et al., 2019; Yamaguchi et al., 2004). Overexpression of *OsYABBY1* leads to extra carpels and stamens (Jang et al., 2004). Another report reveals that *OsYABBY1* determines rice height *via* the feedback regulation of GA biosynthesis (Dai et al., 2007a). *OsSH1* (*OsYABBY2*) is reported that a fragment (>4 kb) insertion in intron three causes loss of *OsSH1* function (Lin et al., 2012). Likewise *OsYABBY1*, overexpression of *OsYABBY4* also leads to a semi-dwarf phenotype by negatively controlling a GA biosynthetic gene, *GA20ox2* (Liu et al., 2007; Yang, Ma & Li, 2016). *OsYABBY5* (formerly known as *OsYAB3*) is negatively regulated by *OsWOX3*, simultaneously repressing the class I *KNOX* (*KNOTTED-LIKE HOMEOBOX*) gene expression in rice leaf development. *OsYABBY5* RNAi plant exhibits a twisted and knotted leaf phenotype (Dai et al., 2007b). Apart from this, *TOB1* (*TONGARI-BOUSHI1*, *OsYABBY5*) is a pleiotropic factor of rice spikelet development that function by a non-cell autonomous manner, and its homolog *TOB2* (*OsYABBY4*), *TOB3* (*OsYABBY3*) also regulate the maintenance and fate of all reproductive meristems (Tanaka et al., 2012; Tanaka, Toriba & Hirano, 2017). Although the function of some *OsYABBYs* have been identified, there are few reports on the regulatory networks and interacting proteins of OsYABBYs.

Prediction and characterization of protein-protein interactions (PPIs) can improve our knowledge of the functions and the 3D structures of proteins, and is essential for proteomics. In this study, the construction of PPI network of OsYABBYs was viewed as an important research content, which will help to understand OsYABBYs transcriptional regulatory network, clarify the interaction between TRs, and provide more accurate target information for rice growth and development.

## Materials and Methods

### Sequence alignment and phylogenetic analysis

YABBY sequences of rice, Arabidopsis, tomato (*Solanum lycopersicum*) and maize (*Zea mays*) were obtained from the Rice Annotation Project Database (https://rapdb.dna.affrc.go.jp/) (Sakai et al., 2013), the TAIR10 database (https://www.arabidopsis.org/) (Lamesch et al., 2012), the SOL Genomics Network (https://solgenomics.net/) (Fernandez-Pozo et al., 2015), and the MaizeGDB database (https://www.maizegdb.org/) (Portwood et al., 2019), respectively. Accession numbers of *YABBY*s were listed in Table S1. Multiple sequence alignment was performed using ClustalX (Larkin et al., 2007) with default values. Then neighbor-joining (NJ) phylogenetic tree and maximum-likelihood (ML) tree were constructed using MEGA7.0 (Kumar, Stecher & Tamura, 2016). For NJ tree construction, the parameters were set to the poisson model, 1,000 bootstrap replicates, and the bootstrap value more than 50% was listed at the branches, and those for ML tree are Jones-Taylor-Thornton (JTT) Model.

### Gene structure, motifs and conserved domains prediction

Exon-intron structures of *OsYABBY*s were confirmed from the Rice Annotation Project Database (Sakai et al., 2013), and the conserved domains of OsYABBYs were identified by the uniprot database (https://www.uniprot.org/*)* (MacDougall et al., 2020). MEME website (https://meme-suite.org/meme/tools/meme) (Nystrom & McKay, 2021) was used to predict the motifs of OsYABBYs with the following parameter settings: the maximum number of motifs, 10; the minimum width and maximum width of motifs, 5 and 20; and default parameters. Eventually, gene structures, motifs, and conserved domains were visualized by IBS1.0.3 (Liu et al., 2015).

### Plant growth conditions, RNA extraction and qRT-PCR

Rice variety Zhonghua 11 (ZH11, *Oryza sativa* ssp *Japonica/geng*) was cultivated in plant growth room. Seven-day-old seedlings were used for sampling. Roots, stems, and leaves were taken followed by a quick grind in liquid nitrogen. Similarly, reproductive tissues like spikelet (40–50 mm), embryo (7–10 days after pollination), stamen (1 day before flowering), pistil (1 day before flowering), palea/lemma (1 day before flowering) at reproductive stage were sampled and immediately stored at −80 °C. Total RNA from different tissues was isolated by TransZol (Transgen ET101-01; TransGen Biotech Co., Ltd., Beijing, China) reagent, and detected by NANODROP 1000 spectrophotometer to determine the extraction quality and concentration, then reversed transcribed into cDNA with M-MLV reverse transcriptase (Thermo Fisher Scientific, Waltham, MA, USA) following the manufacturer’s instructions. Specific primers for all *OsYABBY*s were designed using online programs (https://sg.idtdna.com/scitools/Applications/RealTimePCR/) (Table S2). The RT-qPCR was performed on the QuantStudio 6 Flex real-time PCR instrument (Applied Biosystems, Foster City, CA, USA) using TB Green® Premix Ex Taq™ II reagent (Takara RR820A; Takara, Kusatsu, Japan). *OsActin1* was used as an internal reference to standardize gene expression levels, and each cDNA was subjected to three biological replicates. Relative expression values were calculated using the 2^−ΔΔCt^ method (Livak & Schmittgen, 2001) and the heatmap of gene expression levels was drawn using TBtools (Toolbox for Biologists) v1.09876 software (Chen et al., 2020a).

### PPIs network prediction

OsYABBY interaction proteins were predicted by STRING website (https://string-db.org) (Szklarczyk et al., 2021). Predicted interacting protein score was set to a minimum of 0.4, predicted number of direct interacting proteins was set to no more than 50, and the number of secondary interacting proteins was set to zero. Interaction information was visualized by Cytoscape 3.7.1 (Shannon et al., 2003). Nodes represented proteins, the connections between nodes were represented by edges, which hinted the interactions between these biological molecules (Bader & Hogue, 2003).

### Molecular docking simulation

Based on PPI prediction of interacting proteins, RGAP (Rice Genome Annotation Project) database (http://rice.uga.edu/index.shtml) (Kawahara et al., 2013) was used to obtain protein sequences, and AlphaFold website (https://www.alphafold.ebi.ac.uk/) (Varadi et al., 2022) was used to get PDB files containing 3D structure information. ZDOCK SERVER (http://zdock.umassmed.edu/) (Chen, Li & Weng, 2003) was applied to dock prediction between two proteins by inputting the PDB files. The spatial structures were visualized by PyMOL (Yuan et al., 2016), and the closest distance between possible interacting proteins was measured by the measurement plug-in.

### Yeast two-hybrid

The full length of *OsYABBY* cDNA was amplified and cloned into the bait vector (pGBKT7), and the CDS of *OsWOX3A* and *OsWUS* were obtained and cloned into prey vector (pGADT7). The fused pGBKT7 and pGADT7 vectors were transformed into yeast cells (*AH109*). Transformed cell growth status on the SD/-Leu/-Trp/-His/-Ade medium was used to determine whether the proteins interact with each other. The primers used to construct pGBKT7 and pGADT7 vectors were listed in Table S2.

### Luciferase complementation imaging assay (LCI)

OsYABBYs were fused with the amino-terminal of luciferase to construct the N-luc vector, and OsYABBY-interacted protein coding genes were fused with the carboxyl-terminal of luciferase to construct the C-luc vector. The two fusion proteins were simultaneously expressed in tobacco (*Nicotiana benthamiana*) through agrobacterium-mediated transformation. If the two proteins interacted with each other, the two parts of luciferase would be recombined into whole luciferase, which could oxidate luciferin and generate bioluminescence detected by Tanon5200 (Sun, Zheng & Zhu, 2017).

## Results

### Phylogenetic analysis of YABBY family proteins

**Figure 1:**
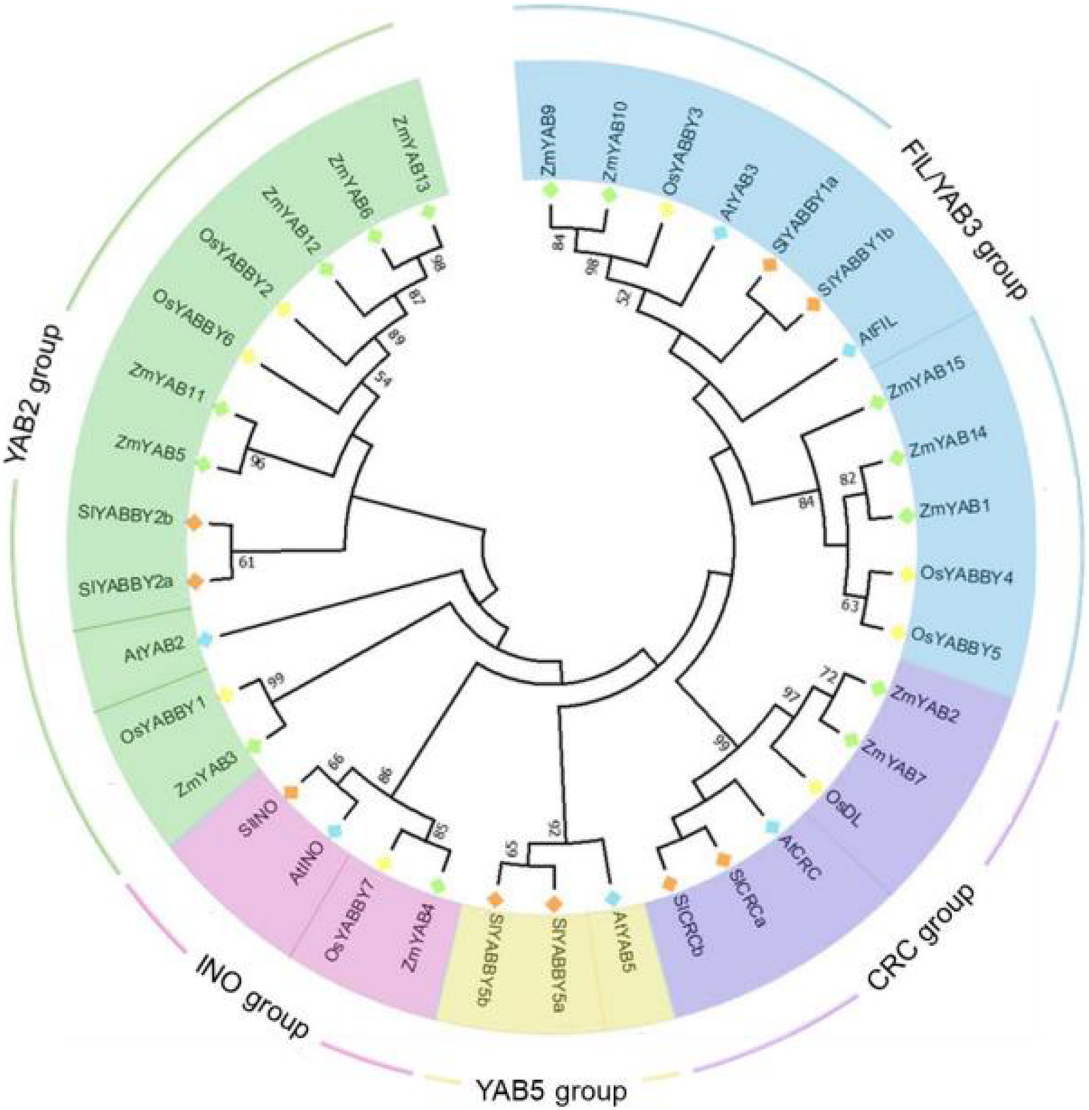
Phylogenetic tree of YABBY proteins.

To reveal the phylogenetic relationship of YABBY proteins, amino acid sequences of Zinc finger and YABBY domains of 37 YABBYs, including eight from rice, six from Arabidopsis, nine from tomato and 14 from maize were aligned and constructed two phylogenetic trees. In NJ (neighbor-joining) tree (Fig. 1), all these YABBYs from four species were clearly divided into five groups: FIL/YAB3, CRC, YAB5, INO and YAB2, based on the similarity of amino acid sequences. OsYABBYs existed in four groups excluding YAB5 group, and ZmYABBYs was also not in YAB5 group, which was consistent with the phylogenetic analysis of other monocot YABBYs (Romanova et al., 2021; Jie et al., 2022) and suggested that the members in YAB5 group might had unique function of dicotyledons. In both CRC and INO groups, dicotyledonous and monocotyledonous YABBYs clustered into a subgroup, respectively, indicating that the function of these YABBYs might have obvious differentiation. However, the differentiation of dicotyledonous and monocotyledonous YABBYs in the FIL/YAB3 and YAB2 groups was not as significant as that in CRC and INO groups. In the ML tree (Fig. S1), similar results to the NJ tree was shown, indicating that the classification of YAB proteins of the NJ tree was reliable.

### Gene structure, motif and conserved domain identification

**Figure 2:**
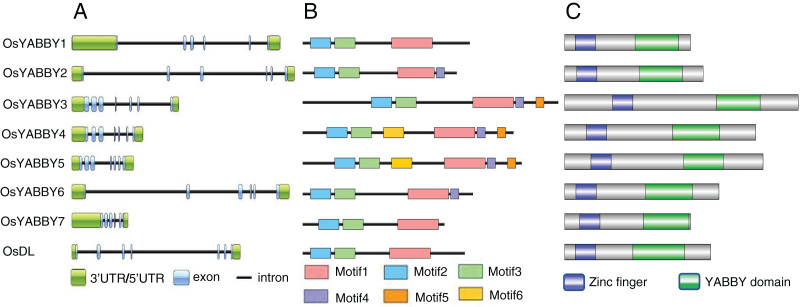
(A-C) Gene and protein structure of OsYABBYs.

Gene structure can provide effective information for understanding gene evolution and function. Structural analysis revealed that FIL/YAB3 and CRC group genes (*OsYABBY3/4/5*, *DL*) contained seven exons, INO and YAB2 group genes (*OsYABBY7*, *OsYABBY1/2/6*) had six exons (Fig. 2A). Members of the same group were more similar to each other than to members of other groups, indicating that each group of *OsYABBY*s might have specific functions.

The function of a protein is determined by its domains, so the motifs and domains of OsYABBYs were analyzed. Six motifs were predicted among all YABBY proteins (Fig. 2B, Fig. S2). OsYABBYs in the same group had similar motifs. For example, YABBY3/4/5 all had motif5, but other YABBYs did not. It might be a motif unique to FIL/YAB3 group (Fig. 2B). Motif1 and motif4 constituted the YABBY domain of OsYABBYs, and motif2 and motif3 constituted the zinc finger domain (Fig. 2C). Both the zinc finger domain and the YABBY domain were considered to be responsible for binding DNA, in which YABBY domain was the main binding site and was also required for YABBY’s nuclear localization (Gross, Broholm & Becker, 2018). However, not every OsYABBY had motif4. Absence of motif4 in some OsYABBYs suggested that the sequences of YABBY domain might be different. Changes in sequence often lead to changes in function. In the site directed mutagenesis of AtCRC, it was found that mutations in C2C2 zinc finger domainat affected the DNA-binding ability, and mutations in YABBY domain destroyed 3D structure and DNA-binding ability (Gross, Broholm & Becker, 2018). Within the amino acid alignment of the domains between OsYABBYs and AtCRC (Fig. S3), it could be seen that a few sites mutated in YABBY domain, suggesting that OsYABBYs might have different DNA-binding ability.

### Expression level of OsYABBYs in various tissues

**Figure 3:**
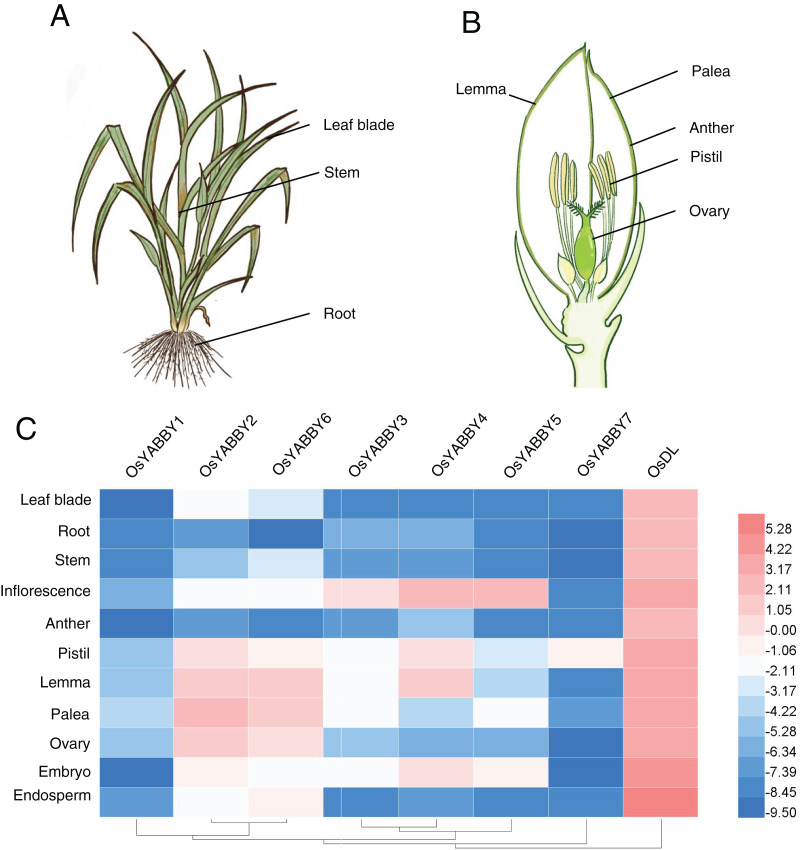
(A-C) Expression profile of OsYABBYs.

Plant-specific TRs, such as *OsWOX11* (Zhao et al., 2009), *OsLBD6* (*OsIG1*) (Zhang et al., 2015), *OsIDD10* (Xuan et al., 2013), play vital roles during organogenesis and exhibit specific expression profiles. The expression data of *OsYABBY*s in various tissues (Figs. 3A and 3B) provided by the RiceXpro website (http://ricexpro.dna.affrc.go.jp/) were used to construct a heat map (Fig. 3C, Table S3). Except for the constitutive expression of *DL*, most *OsYABBYs* were predominantly expressed in reproductive tissues (Fig. 3C).

**Figure 4:**
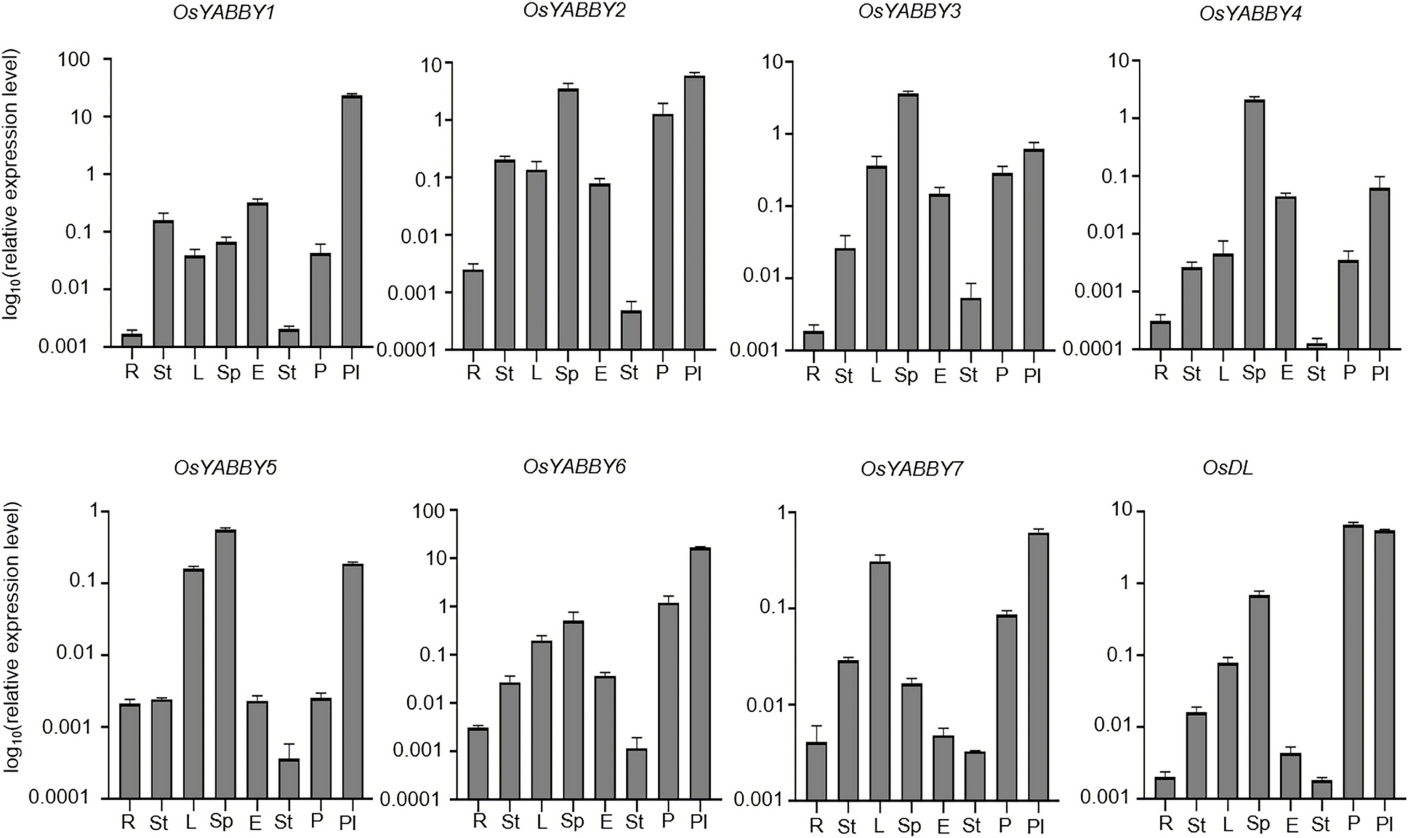
Expression analysis of OsYABBYs in various tissues detected by RT-qPCR.

To verify the reliability of the microarray data of the RiceXpro website, transcriptional level of *OsYABBY*s in vegetative and reproductive organs was detected by RT-qPCR (Fig. 4). All *OsYABBY*s were expressed at a higher level in reproductive organs (spikelet, embryo, stamen, palea/lemma and pistil) than those in vegetative organs (root, stem and leaf). *OsYABBY1/2/6* (YAB2 group) and *OsYABBY7* (INO group) displayed high expression in palea/lemma, suggesting these genes might redundantly regulate palea/lemma development. *OsYABBY1* expressed widely in various tissues, which was in accordance with previous report (Dai et al., 2007b) and indicated *OsYABBY1* might participate in different developmental processes. FIL/YAB3 group genes (*OsYABBY3/4/5*) had higher transcript in spikelet and weaker in root, demonstrating that they might co-regulate spikelet growth. Different from the constitutive expression of *OsDL* in microarray data, RT-qPCR analysis showed tissue-specific expression pattern of *OsDL*, which was mainly expressed in pistil, palea/lemma and spikelet. These results suggested that different *OsYABBY*s might be responsible for the development of different reproductive tissues.

### Prediction and verification of OsYABBY interacting proteins

In order to further elucidate OsYABBY functions, the interacting proteins of OsYABBYs were investigated through STRING website (https://string-db.org). The results revealed that some OsYABBYs could interact with each other (Fig. S4), and also form complex with other proteins (Fig. S5). Since the complexes that formed between YABBYs had been reported (Sieber et al., 2004; Stahle et al., 2009), the more other proteins that could interact with YABBYs needed to be elucidated. As TRs, YABBYs function in the nucleus, their interaction candidate proteins should also be localized in the nucleus. Based on this standard, 17 non-YABBY proteins were screened, including transcription factors (TFs), chromosome recombination factors, elongator-associated factor, zinc transporter, gibberellin regulatory protein, enzymes and chaperone (Fig. S5A, Table S4). Among these, the number of TFs accounted for 70% of all proteins, and the WOX (WUSCHEL-related homeobox) family proteins accounted for 30% of TFs, indicating that OsYABBYs and OsWOXs might interact with each other (Fig. S5B). It was reported that some *OsWOX* genes (*OsWUS*, *OsWOX3A*, *OsWOX9A*) were highly expressed in reproductive organs and function in reproductive organ development (Cho et al., 2013; Cheng et al., 2014), which was similar to expression pattern and role of *OsYABBY*s. Based on interaction prediction and expression profiling analysis of *OsWOXs* and *OsYABBY*s, interaction verification of OsYABBYs and OsWOXs was further conducted by molecular 3D docking, Y2H and LCI experiments.

**Figure 5:**
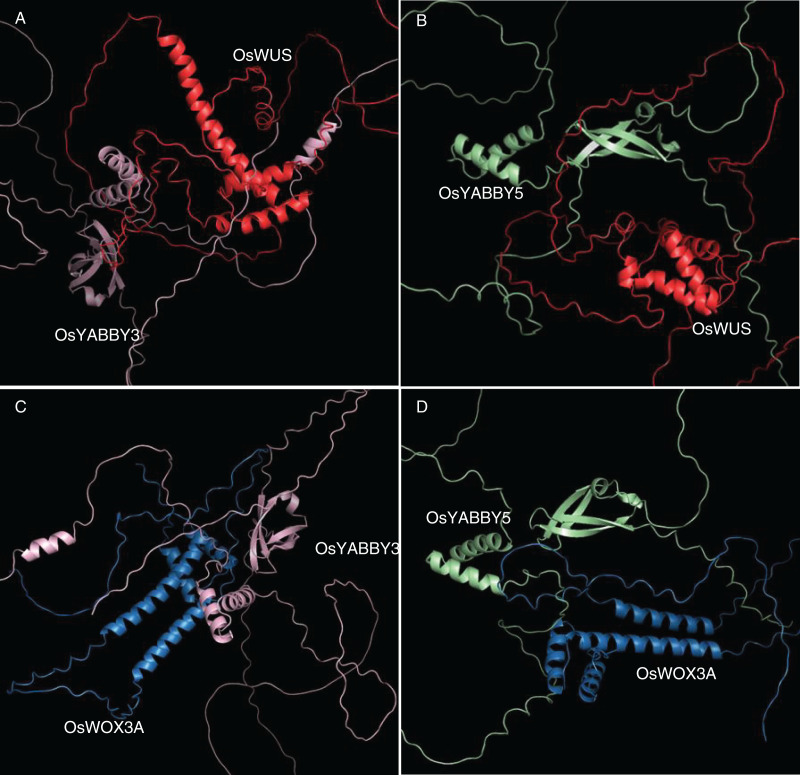
(A-D) Tertiary structure simulation and molecular docking.

The 3D docking of OsYABBYs and OsWOXs was executed by ZDOCK program, which was a docking program to predict several protein complexes *via* PSC (Pairwise Shape Complementarity) of input protein structure. The docking results were visualized by PyMOL software (Fig. 5), and the closest distances measured by PyMOL and the closest distances between hydrogen bonds were organized into Table S5. Two groups of proteins YABBY3-WUS and YABBY5-WUS were the closest in space, with distances of 1.0 and 0.9 Å, respectively. The closest distances of YABBY3-WOX3A and YABBY5-WOX3A are 3.2, and 3.1 Å, respectively. The reliability of the STRing website results was confirmed by the distance between proteins, and the information between hydrogen bonds can also predict these protein interactions.

**Figure 6:**
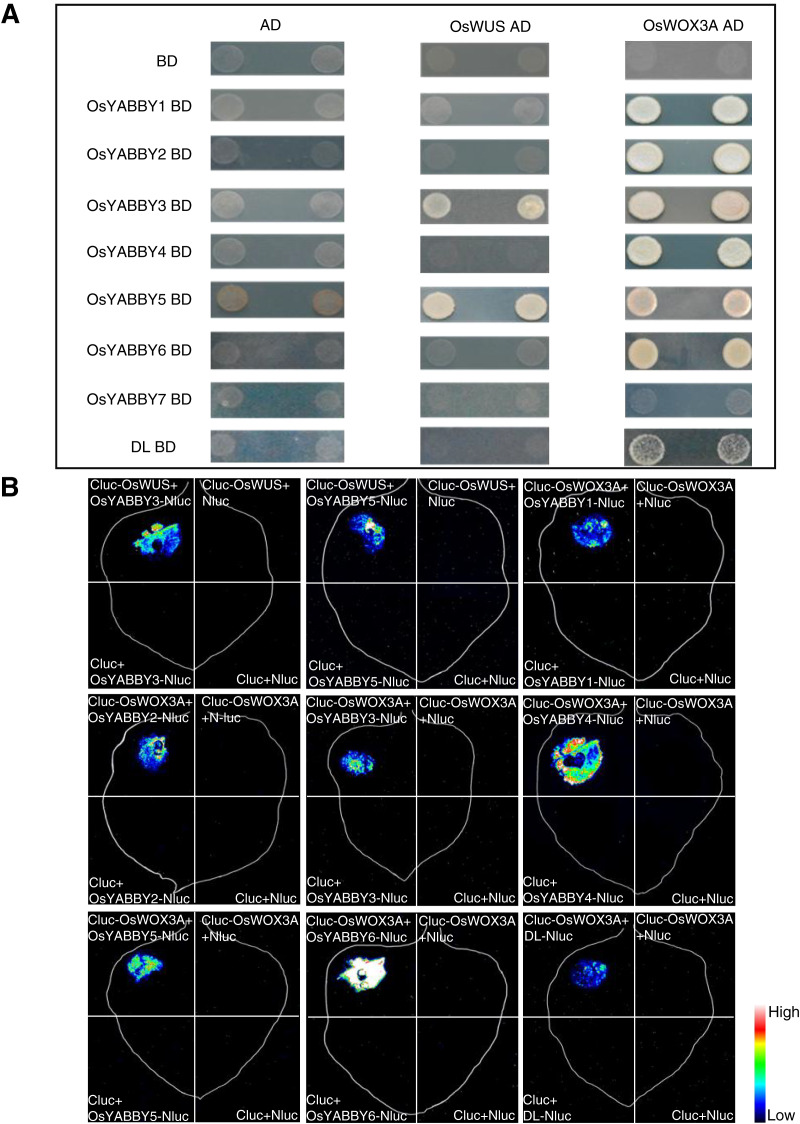
The interaction of OsYABBYs with OsWUS and OsWOX3A. (A) Protein interaction identified by Y2H. AD and BD indicated empty pGADT7 and pGBKT7 vectors, respectively. SD/-Ade/-His/-Leu/-Trp indicated SD medium lacking Ade, His, Leu, and Trp. (B) Protein interaction identified by LCI. Cluc vector and Nluc vector were selected as negative control. The pseudocolor bars indicated the range of luminescence intensity in each image.

The Y2H suggested that OsYABBYs except OsYABBY7 could interact with OsWOX3A, and OsYABBY3, OsYABBY5 could interact with OsWUS (Fig. 6A). LCI experiment further verified the results of Y2H (Fig. 6B). It could be seen from the Y2H and LCI that OsYABBY5-OsWUS, OsYABBY4-OsWOX3A, OsYABBY6-OsWOX3A had strong interactions, while DL-OsWOX3A had weaker interaction (Fig. 6). The results showed that there was indeed possibility of interaction between OsYABBYs and OsWOXs, which would lay a foundation of interpreting OsYABBYs regulatory mechanisms.

## Discussion

YABBYs, both C2C2 zinc finger and YABBY domain-containing TRs, are widely involved in lateral organ development in higher plants and the identification of YABBY gene family has made progress in many plants (Lu et al., 2021; Xia et al., 2021; Yin et al., 2022; Zeng et al., 2022). However, systematic investigation of the OsYABBYs-interacting proteins have been poorly reported. In present study, OsYABBYs evolutionary relationship, gene structures, conserved domains, and expression pattern were analyzed. More importantly, their interaction protein information was determined.

It has been reported that YABBY genes have shown functional differentiation in some species, such as *Phalaenopsis orchid, Brassica napus*, *Cucumis sativus* (Chen et al., 2020b; Xia et al., 2021; Yin et al., 2022), and so on. According to the clustering and expression pattern of this study, OsYABBYs also exhibited functionally diverged and were involved in different biological processes. In the YAB3 group, *OsYABBY3*, *OsYABBY4* and *OsYABBY5* were proved to affect spikelet development (Tanaka, Toriba & Hirano, 2017), which was accordance with their expression profiles (Fig. 4). In the CRC group, *OsDL* was highly expressed in pistil (Fig. 4) and in line with its reported regulation of carpel specification (Yamaguchi et al., 2004). In the YAB2 group, *OsYABBY1* was confirmed to control carpel and stamen development, and plant height, due to its wide expression trait (Fig. 4). These results indicated that OsYABBYs in different groups had different function based on their various expression pattern, which suggested that expression profile of *OsYABBY*s might one major reason of their function divergence.

Protein interactions also play an essential role in protein functional differentiation. In *Arabidopsis*, YABBY-interacting proteins had been identified and characterized by biochemical and genetic methods. For instance, LUG, LUH and LUG-associated coregulator SEUSS were verified as interacting proteins of most vegetative AtYABs (FIL, YAB3, and YAB5) (Stahle et al., 2009; Boter et al., 2015). NOZZLE (NZZ) was confirmed to bind FIL, YAB3 and INO, in which INO was one of reproductive AtYABs (Sieber et al., 2004). In rice, OsSEU3 was indicated as OsYABBY5 interaction protein, co-regualting the development of rice floral organs (Tanaka, Toriba & Hirano, 2017). And interacting proteins with other OsYABBYs are rarely reported. In this study, 17 non-YABBY proteins in the STRING database were predicted to be OsYABBY interacting proteins. No rice homologs of reported AtYABBY interacting proteins that had been reported were found in the 17 proteins, which suggested that difference of YABBY interacting proteins might be responsible for function divergence between monocotyledonous and dicotyledonous YABBYs. Among predicted interacting proteins of OsYABBYs, members of OsWOX family accounted for a relatively high proportion, and shared similar expression pattern and function with OsYABBYs, such as severely defective in tillering and flower development in *oswus* mutant (Tanaka et al., 2015), and narrow-curly leaves, increased tillers, abnormal development of floral organs in *oswox3a* mutant (Cho et al., 2013; Yoo, Cho & Paek, 2013). In addition, the assay of OsYABBY-OsWOX docking suggests the possibility of interaction between them (Fig. 5). And the evidence of interaction between DoYABBY and DoWOX in *Dendrobium candidum* further supports our prediction (Zeng et al., 2022). Based on these results, we speculated that the interaction between OsYABBY and OsWOX family members might be a major regulatory pathway for its function. Furthermore, we found that the OsYABBYs of different groups exhibited different interacting network by interacting with different proteins. The YAB2 group (OsYABBY1/2/6) and the FIL/YAB3 group (OsYABBY3/4/5) could interact with OsWOX3A, but only the FIL/YAB3 group members (OsYABBY3/5) could interact with OsWUS, and the CRC group (DL) and the INO group (OsYABBY7) had no or weak ability to interact with OsWUS and OsWOX3A (Fig. 6). The difference in interaction results suggested that OsYABBYs of different groups might achieve their functions through different pathways, which also hinted that it might be another reason for OsYABBY’s function divergence and paved the way for our next step in studying the function of OsYABBYs.

As a plant-specific TR, the regulatory mechanism of OsYABBYs have not been analyzed in detail. In previous reports, WOX family members were thought to be downstream of YABBYs (Nakata et al., 2012), but our study reveals OsWOX maybe improtant interacting proteins of OsYABBYs, which laid foundation for explaining the regulatory network of YABBY. Furthermore, YABBY domain was regarded as high similarity with HMG-box domain (Filyushin et al., 2017; Gross, Broholm & Becker, 2018), and whether OsYABBYs could function as chromatin proteins also need further study. Overall, our study provides guidance and direction for future research of OsYABBYs.

## Conclusions

Eight OsYABBYs were classified into four groups based on protein sequences. Gene structure, domain analysis and expression pattern demonstrated the structural and functional differentiation of OsYABBYs. Public microarray datasets and RT-qPCR data revealed that *OsYABBY*s had tissue/organ-specific expression profiles. Importantly, PPIs analysis revealed OsYABBYs could interact with OsWUS and OsWOX3A, which would provide a theoretical basis for further analysis of OsYABBYs.

## Supplemental Information

10.7717/peerj.14783/supp-1 Supplemental Information 1 Gene information of YABBY genes in rice, Arabidopsis, maize and tomato. Loc numbers of all genes used in the phylogenetic tree.

10.7717/peerj.14783/supp-2 Supplemental Information 2 Primer sequence information used in this study.

10.7717/peerj.14783/supp-3 Supplemental Information 3 The expression levels of OsYABBY genes derived from microarray data.

10.7717/peerj.14783/supp-4 Supplemental Information 4 Information about proteins in interaction networks.

10.7717/peerj.14783/supp-5 Supplemental Information 5 Molecular distance prediction.

10.7717/peerj.14783/supp-6 Supplemental Information 6 Maximum-likelihood (ML) phylogenetic tree tree of YABBY proteins.

10.7717/peerj.14783/supp-7 Supplemental Information 7 Motif sequences of OsYABBYs detecting by MEME.

10.7717/peerj.14783/supp-8 Supplemental Information 8 Sequence alignment of Zinc finger domain (A) and YABBY domain (B) between OsYABBYs and AtCRC.

10.7717/peerj.14783/supp-9 Supplemental Information 9 STRING predicted interaction network between OsYABBYs.

10.7717/peerj.14783/supp-10 Supplemental Information 10 Interaction between OsYABBYs and other proteins.

10.7717/peerj.14783/supp-11 Supplemental Information 11 RT-qPCR data: YABBYs expression data in root, leaf, pistol, and stamen.

10.7717/peerj.14783/supp-12 Supplemental Information 12 RT-qPCR data: YABBYs expression data in stem, spikelet, and palea/lemma.

10.7717/peerj.14783/supp-13 Supplemental Information 13 RT-qPCR data: YABBYs expression data in embryo.

## Additional Information and Declarations

### Data Availability

The following information was supplied regarding data availability: The raw measurements of RT-qPCR are available in the Supplemental Files.
