# Supplementary material for: Comprehensive study of rice YABBY gene family: evolution, expression and interacting proteins analysis
Source: PeerJ. 2023 Feb 24;11:e14783. doi: 10.7717/peerj.14783 (PMC9969854; doi:10.7717/peerj.14783)
Supplement: Supplemental Information 6 [file peerj-11-14783-s006.pdf]

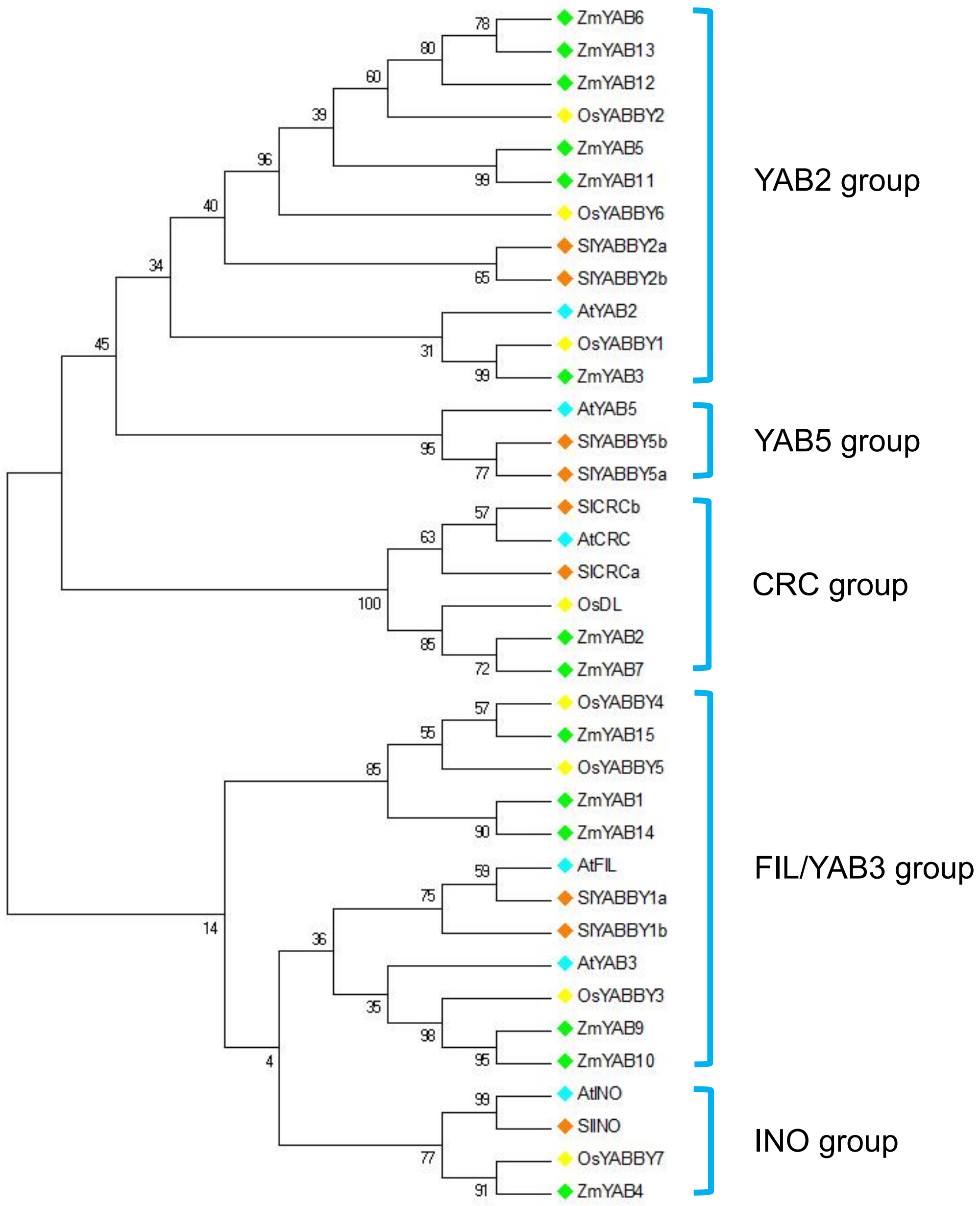

Figure S1 Maximum-likelihood (ML) phylogenetic tree tree of YABBY proteins involving *Oryza sativa* (Os), *Arabidopsis thaliana* (At), *Solanum lycopersicum* (Sl) and *Zea mays* (Zm).
