# Supplementary figures and images for: Comprehensive study of rice YABBY gene family: evolution, expression and interacting proteins analysis
Source: PeerJ. 2023 Feb 24;11:e14783. doi: 10.7717/peerj.14783 (PMC9969854; doi:10.7717/peerj.14783)

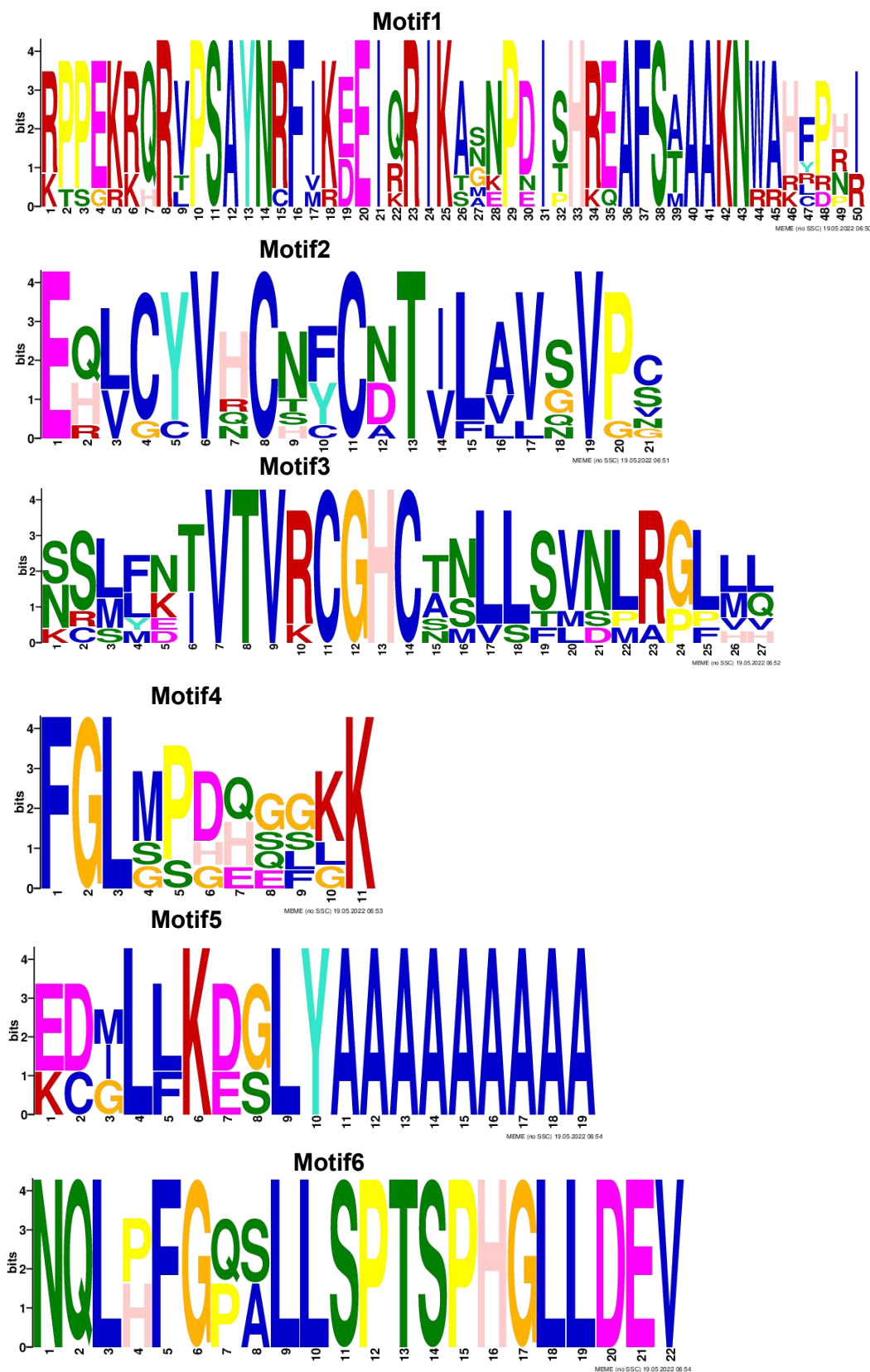

**Figure S2** Motif sequences of OsYABBYs detecting by MEME.

Supplement: Supplemental Information 7 [file peerj-11-14783-s007.pdf]

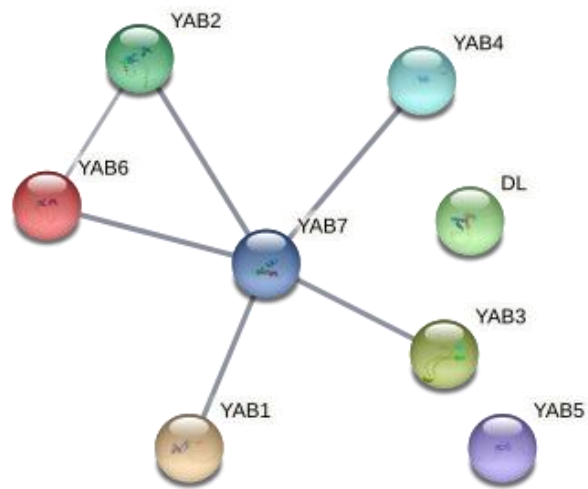

**Figure S4** STRING predicted interaction network between OsYABBYs.

Supplement: Supplemental Information 9 [file peerj-11-14783-s009.pdf]
