# Supplementary material for: Comprehensive study of rice YABBY gene family: evolution, expression and interacting proteins analysis
Source: PeerJ. 2023 Feb 24;11:e14783. doi: 10.7717/peerj.14783 (PMC9969854; doi:10.7717/peerj.14783)
Supplement: Supplemental Information 8 [file peerj-11-14783-s008.pdf]

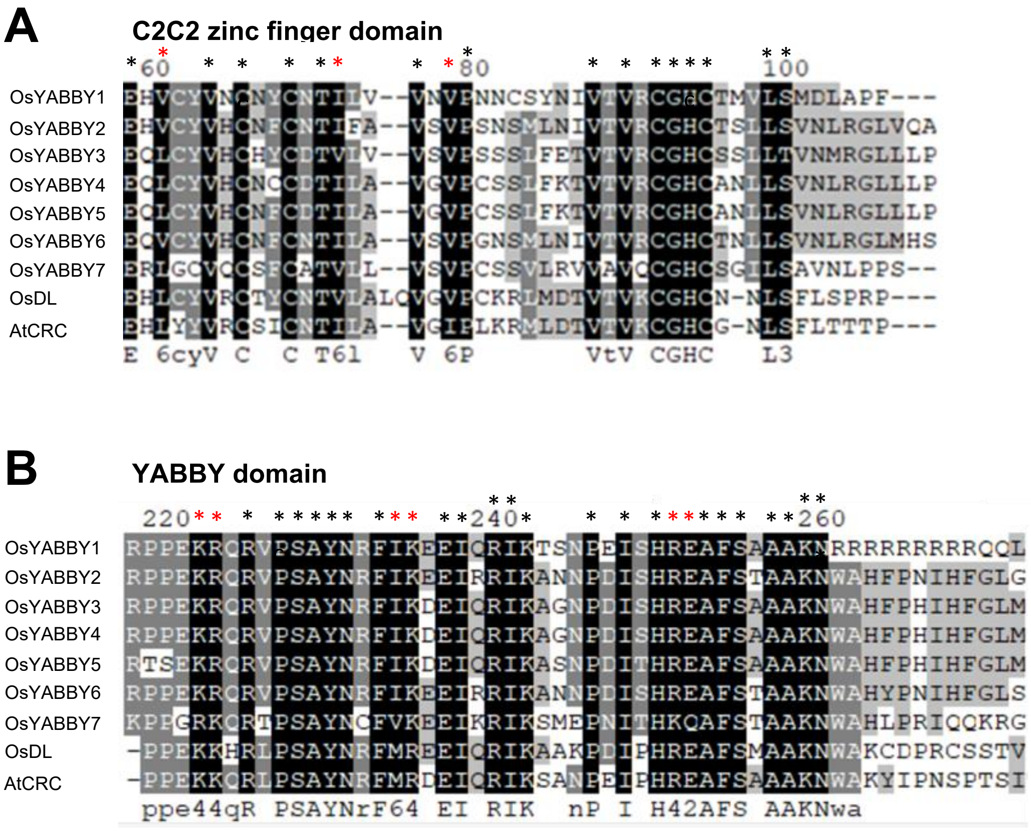

Figure S3 Sequence alignment of Zinc finger domain (A) and YABBY domain (B) between OsYABBYs and AtCRC. Asterisks indicate important amino acid positions in the domain, black and red asterisks showed unchanged and changed amino acid sites, respectively.
