# Supplementary material for: Comprehensive study of rice YABBY gene family: evolution, expression and interacting proteins analysis
Source: PeerJ. 2023 Feb 24;11:e14783. doi: 10.7717/peerj.14783 (PMC9969854; doi:10.7717/peerj.14783)
Supplement: Supplemental Information 10 [file peerj-11-14783-s010.pdf]

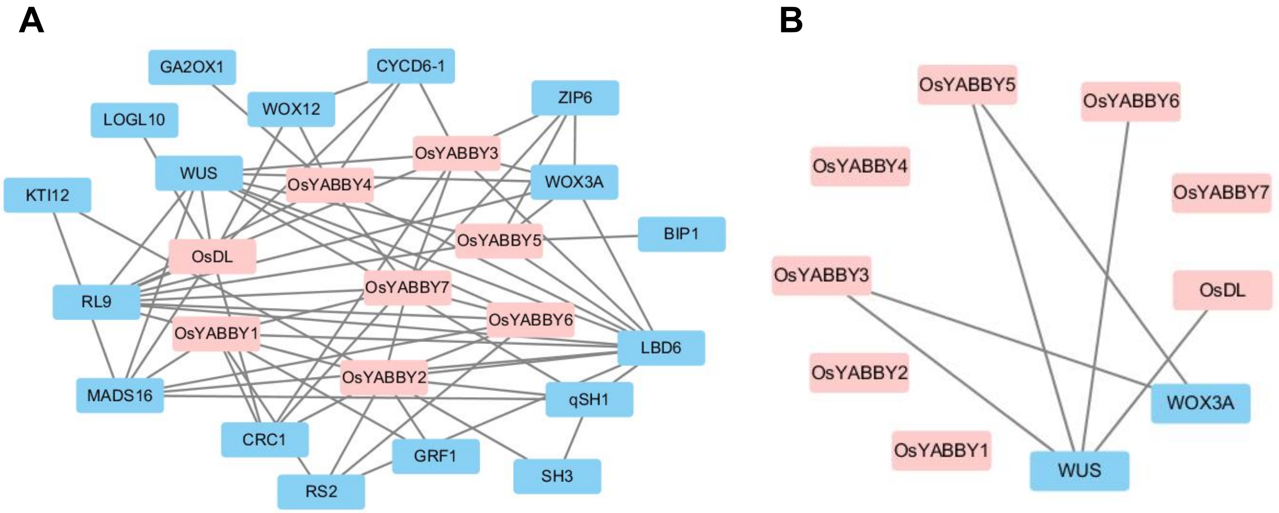

Figure S5 Interaction between OsYABBYs and other proteins. (A) PPI network predicted by the STRing website. (B) The interaction between OsYABBY and WUSCHEL-LIKE HOMEOBOX 1 (WUS) and WUSCHEL-related homeobox 3A (WOX3A). The pink and blue nodes represented OsYABBY proteins and their interacting proteins, respectively.
